# Supplementary material for: Taxonomic revision of the tarantula genus Aphonopelma Pocock, 1901 (Araneae, Mygalomorphae, Theraphosidae) within the United States
Source: Zookeys. 2016 Feb 4;(560):1–340. doi: 10.3897/zookeys.560.6264 (PMC4768370; doi:10.3897/zookeys.560.6264)
Supplement: Supplementary material 2 — Compressed files (.zip) containing Aphonopelma boxplot and PCA morphospace data. [file zookeys-560-001-s002.zip › Supp2b.zip!/Appendix 2B/Boxplot_morphospace/Aphonopelma_boxplots_females/ALL/P3M3_ratio_females.pdf]

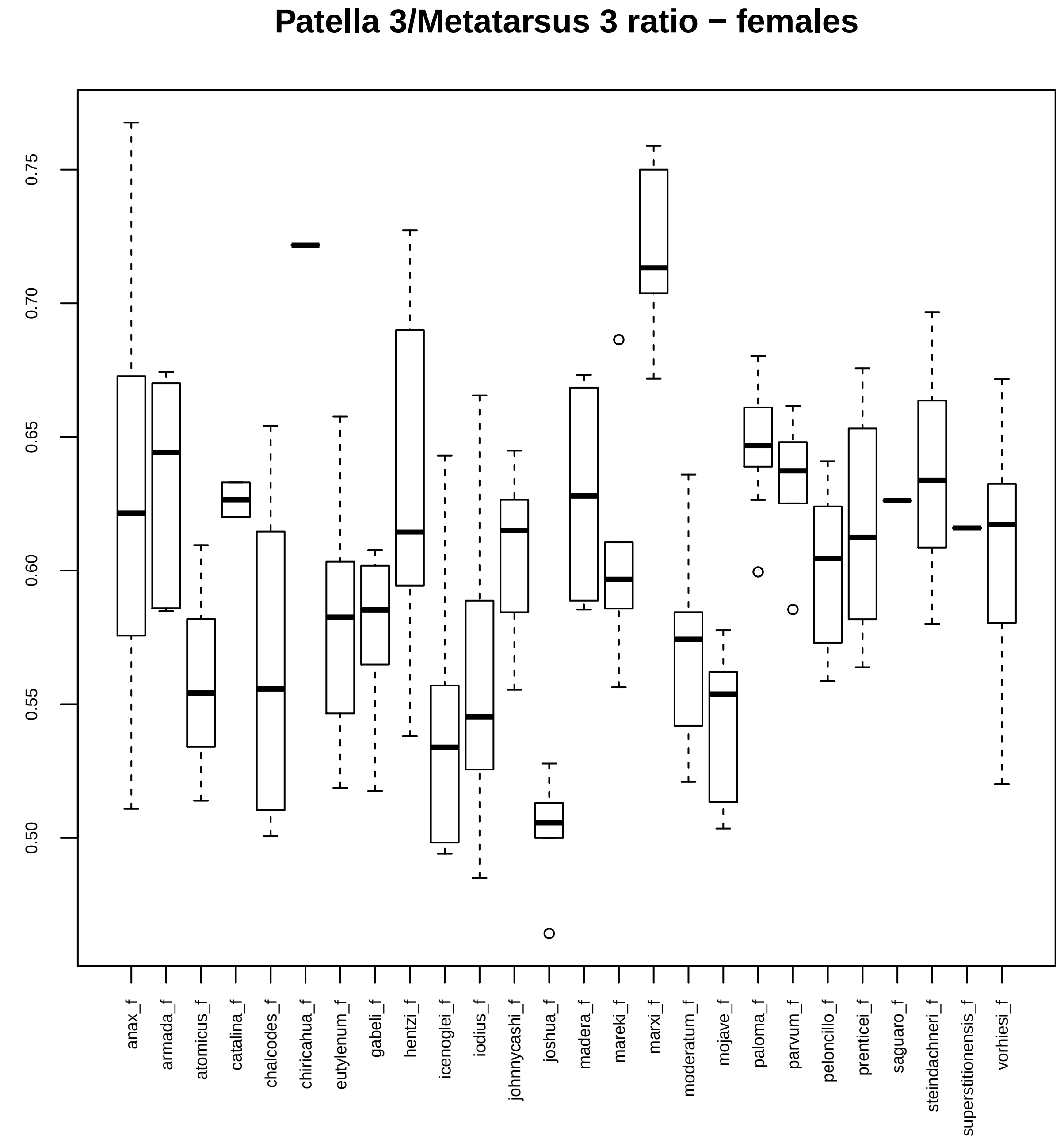
Patella 3/Metatarsus 3 ratio − females
0.50
0.55
0.60
0.65
0.70
0.75
anax_f
armada_f
atomicus_f
catalina_f
chalcodes_f
chiricahua_f
eutylenum_f
gabeli_f
hentzi_f
icenoglei_f
iodius_f
johnnycashi_f
joshua_f
madera_f
mareki_f
marxi_f
moderatum_f
mojave_f
paloma_f
parvum_f
peloncillo_f
prenticei_f
saguaro_f
steindachneri_f
superstitionensis_f
vorhiesi_f
